# Association of Epstein-Barr virus antibody titers with a human IL-10 promoter polymorphism in Japanese women

**DOI:** 10.1186/1740-2557-5-2

**Published:** 2008-03-04

**Authors:** Yutaka Yasui, Nobuyuki Hamajima, Tsuneya Nakamura, Noha Sharaf El-Din, Kazuo Tajima, John D Potter

**Affiliations:** 1 Department of Public Health Sciences, School of Public Health, University of Alberta, Edmonton, AB, Canada; 2 Department of Preventive Medicine, Biostatistics, and Medical Decision Making, Nagoya University Graduate School of Medicine, Nagoya, Japan; 3 Department of Gastroenterology, Aichi Cancer Center Hospital, Nagoya, Japan; 4 Division of Epidemiology and Prevention, Aichi Cancer Center Research Institute, Nagoya, Japan; 5 Division of Public Health Sciences, Fred Hutchinson Cancer Research Center, Seattle, WA, USA

## Abstract

**Background:**

Multiple sclerosis (MS) risk, over 10-fold higher in Western than in Asian countries, is associated with elevated IgG antibody titers against Epstein-Barr viral capcid antigen (anti-EBVCA IgG titers). Given the 84% homology of the open reading frame BCRF1 of Epstein-Barr virus (EBV) to human interleukin 10 (hIL-10) and the remarkable Caucasian-vs.-Asian population differences in hIL-10 gene promoter polymorphisms, this strong association of MS risk with anti-EB-VCA IgG titers may be explained by the genetic variations in the hIL-10 gene.

**Methods:**

We evaluated anti-EB-VCA IgG titers in association with a single nucleotide polymorphism (SNP) in the promoter of hIL-10 at position -819 (hIL-10 T-819C) in a cross-sectional survey of 241 Japanese. Anti-EB-VCA IgG titer and its elevation (≥ 1:160) were evaluated, stratified by sex and hIL-10 T-819C genotype.

**Results:**

The cytosine-allele frequencies at hIL-10 T-819C were 32.9% in women and 30.9% in men. These are consistent with the published reports of Japanese and Chinese, but substantially lower than those of Caucasians (> 70%). In women, the proportion with elevated anti-EB-VCA IgG titers (≥ 1:160) increased appreciably from 53.7% in the T/T genotype group to 66.7% in the T/C group and to 83.3% in the C/C group (P-trend = 0.037). The titers did not differ by the hIL-10 T-819C genotype in men.

**Conclusion:**

Anti-EB-VCA IgG titers may increase with the number of cytosine alleles at hIL-10 T-819C in women. This observed gender specific association in Japanese warrants further investigation, especially in Western populations with high MS risk.

## Background

Human interleukin 10 (hIL-10) is a pleiotropic cytokine with multifaceted functions in the regulation of immune and inflammatory responses [1]. It limits cell-mediated immune responses by inhibiting the activation of macrophages, monocytes, and dendritic cells. In B cells, hIL-10 promotes cell proliferation and differentiation, and also plays some anti-apoptotic function. These biological activities of hIL-10 are advantageous for viruses that infect and persist in B cells. Indeed, Epstein-Barr virus (EBV), as well as some other members of the herpes virus family, appears to use mimicry of hIL-10 functions as part of their survival strategies in the host. Specifically, an open reading frame of EBV, BCRF1, shows a strikingly high degree of sequence homology with hIL-10 (84% homology in amino acid sequence) and viral IL-10 shares many of hIL-10's biological activities [1]. Based on these observations, we hypothesize that genetic variations in the host's hIL-10 gene may modulate the host-organism interaction with this ubiquitous virus encoding an hIL-10 mimic.

This hypothesis is of particular interest as both the risk of multiple sclerosis (MS) and the cytosine-allele frequency at position -819 in the promoter region of hIL-10 (hIL-10 T-819C) are considerably higher in Western, compared to Chinese and Japanese populations (MS prevalence of < 5 per 100,000 in China and Japan [2] vs. > 50 per 100,000 in the US [3] and the cytosine-allele frequency at hIL-10 T-819C of 28.8–32.7% in Chinese and Japanese [4-7] vs. 71.8–83.6% in Caucasians [7-12]). Furthermore, there is a strong positive association between MS risk and elevated pre-diagnosis IgG antibody titers against Epstein-Barr viral capsid antigen (anti-EBVCA IgG titers) [13-21] although an inverse association has been observed in one prospective study [22].

To test the hypothesis, anti-EB-VCA IgG titers were measured as an indicator of the host-virus interaction, and compared across the genotypes of the hIL-10 promoter single nucleotide polymorphism (SNP) at hIL-10 T-819C.

## Methods

### Study population

A cross-sectional survey was conducted including 241 Japanese outpatients, 123 females and 118 males, aged 39–69, without any history of cancer, who underwent physical examination and gastroscopy at the Aichi Cancer Center Hospital between March and December 1999. Patients with autoimmune diseases were excluded. A written informed consent for providing peripheral blood sample and its analysis for DNA polymorphisms was obtained from each participant.

### IL-10 polymorphism assay and EBV antibody assay

DNA was extracted from buffy coat using a QIAamp blood mini kit (Qiagen, Valencia, CA) and the hIL-10 promoter SNP at position -819 was characterized by the polymerase chain reaction with confronting two-pair primers (PCR-CTPP), which does not require restriction enzyme digestion, developed by our laboratory [23].

Anti-EB-VCA IgG titers were measured using plasma samples by indirect immunofluorescence [24] at a commercial laboratory (Mitsubishi Chemical BCL, Tokyo, Japan). The titer was defined by the maximum dilution with definitely visible fluorescence. Titers at or above the median level (≥ 1:160) were considered to be "elevated." In a separate study with 600 samples, we tested the laboratory's assay reliability by including 30 blinded identical quality-control samples in the 600 samples. The laboratory showed an excellent reliability returning an identical anti-EB-VCA IgG titer for 27 of the 30 samples and the one-dilution lower titer for the remaining 3 samples.

### Statistical analysis

Geometric mean and standard deviation of anti-EB-VCA IgG titers were calculated for each hIL-10 T-819C genotype and stratified by sex. Elevation of anti-EB-VCA IgG titers (≥ 1:160) in relation to the hIL-10 T-819C genotypes was examined by unconditional logistic regression [25] adjusting for age and stratified by sex. The observed genotype groups were T/T, T/C, and C/C. The trend in the proportion of elevated titers across the three genotypes, ordered by the number of cytosine alleles, was examined by Armitage-Cochran Trend Test [26], adjusting for age. All significance tests were two sided.

## Results

Table 1 shows the geometric mean and standard deviation of anti-EB-VCA IgG titers by the hIL-10 T-819C genotype, stratified by sex. The geometric mean titer increased monotonically with the number of cytosine alleles among women, but not among men. In the 123 women, the proportion with elevated anti-EB-VCA IgG titers increased from 53.7% in the T/T group to 66.7% in the T/C group and 83.3% in the C/C groups (P-trend = 0.037) (Table 2). This was not seen among the 118 men (P-trend = 0.84). The difference of the trend between the sexes was marginally statistically significant (P = 0.097).

**Table 1:** Geometric mean and standard deviation of IgG antibody titers against Epstein-Barr viral capsid antigen (anti-EB-VCA IgG titer) by IL-10 T-819C genotype, stratified by sex

IL-10 T-819C genotype	Women		Men	
	
	Total number (%)	Geometric mean anti-EB-VCA IgG titer (×/÷ Geometric standard deviation)	Total number (%)	Geometric mean anti-EB-VCA IgG titer (×/÷ Geometric standard deviation)
T/T	54 (43.9)	1:127 (×/÷ 2.4)	56 (47.5)	1:122 (×/÷ 2.0)
T/C	57 (46.3)	1:152 (×/÷ 2.1)	51 (43.2)	1:140 (×/÷ 2.0)
C/C	12 (9.8)	1:190 (×/÷ 2.1)	11 (9.3)	1:124 (×/÷ 2.7)

**Table 2:** Elevation of IgG antibody titers (≥ 1:160) against Epstein-Barr viral capsid antigen (anti-EB-VCA titer) by IL-10 T-819C genotype, stratified by sex+

IL-10 T-819C Genotype	Women		Men	
	
	Number with anti-EB-VCA IgG titer ≥ 1:160/Total (%)	Odds ratio of anti-EB-VCA IgG titer ≥ 1:160 (95% CI)	Number with anti-EB-VCA IgG titer ≥ 1:160/Total (%)	Odds ratio of anti-EB-VCA IgG titer ≥ 1:160 (95% CI)
T/T	29/54 (53.7)	1.00 (Reference)	32/56 (57.1)	1.00 (Reference)
T/C	38/57 (66.7)	1.72 (0.80–3.75)	32/51 (62.7)	1.26 (0.58–2.76)
C/C	10/12 (83.3)	4.31 (1.01–29.79)	5/11 (45.5)	0.63 (0.16–2.31)
		P-trend* = 0.037		P-trend* = 0.84

*Armitage-Cochran Trend Test, adjusted for age

+Trend difference between sexes: P = 0.097

## Discussion

In this study, we found a monotonic increase in anti-EB-VCA IgG titers with the number of cytosine alleles at the hIL-10 T-819C locus in Japanese women, however not in men. Considering the higher prevalence of both MS [2,3] and the specific SNP in the promoter of hIL-10T-819C [4-12] in Western populations compared to Asian populations, and the strong positive association of MS risk with anti-EB-VCA IgG titers [13-21], our finding has an important implication on MS susceptibility. Specifically, it suggests a genetic predisposition to MS in the hIL-10 gene promoter, relevant only in women.

Such female-specific MS-risk-elevating function is consistent with the notion that gonadal steroids regulate the gender dimorphism in MS and other autoimmune diseases [27]. Indeed, in the mouse model of MS, experimental autoimmune encephalomyelitis in SJL mice, disease severity is increased by castration in male mice [28] and decreased by testosterone implantation in females [29]. The immunomodulatory effects of gonadal steroids, especially, testosterone, may underlie the female-specific association of anti-EB-VCA IgG titers with hIL-10 T-819C observed here.

Studies that have investigated human MS risk in relation to polymorphisms in hIL-10 have found no association [30-37]. These studies, however, investigated Western high-risk populations where the cytosine-allele frequencies at hIL-10 T-819C are very high (> 70%). They did not investigate, with a few exceptions, the polymorphism at hIL-10 T-819C whose genotype distributions are markedly different between the high- and low-risk populations of MS, and did not stratify the association analysis by gender. It is also a possibility that the T-819C polymorphism and the associated IL-10 levels may modulate the immune response and MS risk, given some observed correlation between the promoter polymorphism and the IL-10 levels in *in vitro/in vivo *[38]. It would be of interest to evaluate MS risk in relation to the hIL-10 T-819C genotype: (1) in populations where thymine-allele frequencies at hIL-10 T-819C are sufficiently high (e.g., Chinese and Japanese); and perhaps more importantly, (2) in any population stratified by gender.

Our findings are, however, consistent with a recent study of a small patient cohort showing a statistically significant increase in CC genotype of hIL-10 SNPs at -819 and -592 loci in MS patients [39]. The hIL-10 promoter polymorphism at the -819 locus is often analyzed along with the hIL-10 promoter polymorphisms at the -1082 and -592, which is justified by their strong linkage disequilibrium, in addition to the three main haplotypes (GCC, ACC, ATA, and very rarely GTA, at the -1082, -819, and -592 locus) found to segregate in most populations. Because we did not study the -1082 locus, we were unable to separate the GCC and ACC haplotypes. However, G alleles at the -1082 locus are very rare (G-allele frequency < 4%) in Japanese and Chinese populations [4-7].

## Conclusion

Given the high homology of viral IL-10 to hIL-10, the established association of MS with elevated anti-EB-VCA IgG titers, and the public health/personal burden of MS, our finding of female-specific increase in anti-EB-VCA IgG titers with the number of cytosine alleles at hIL-10 T-819C warrants further investigation. Such investigations may lead to an explanation of high MS risk in Western populations, where the MS disease burden is more than 10 times higher than that in Japan and China, and the cytosine-allele frequencies at hIL-10 T-819C are at least twice as high (> 70% vs. 30%) as those in Chinese and Japanese populations.

## Competing interests

The author(s) declare that they have no competing interests.

## Authors' contributions

NH, TN, KT designed and conducted the epidemiologic study on which this study was based. The original idea of this study was conceived by NH and YY. NH performed the polymorphism analysis. Data analysis was performed by YY. The paper was drafted by YY, revised by JDP and NSED, finalized and approved by all authors.
